# Prevalence of drug-drug interactions in oncology patients enrolled on National Clinical Trials Network oncology clinical trials

**DOI:** 10.1186/s12885-018-5076-0

**Published:** 2018-11-22

**Authors:** Lauren A. Marcath, Taylor D. Coe, Emily K. Hoylman, Bruce G. Redman, Daniel L. Hertz

**Affiliations:** 1 Department of Clinical Pharmacy, University of Michigan, College of Pharmacy, Ann Arbor, MI 48109-1065 USA; 2 Department of Internal Medicine – Hematology/Oncology, University of Michigan, Michigan Medicine, Ann Arbor, MI 48109-1065 USA

**Keywords:** Oncology clinical trial drug interaction

## Abstract

**Background:**

Drug-drug interactions (DDIs) in subjects enrolling in clinical trials can impact not only safety of the patient but also study drug outcomes and data validity. This makes it critical to adequately screen and manage DDIs. The study objective was to determine the prevalence of DDIs involving study medications in subjects enrolling in National Clinical Trials Network (NCTN) clinical trials at a single institution. DDIs were evaluated based on study protocol recommendations for concomitant medication use (i.e. exclude, avoid or use caution), screening via DDI tool, and pharmacist review.

**Methods:**

Subjects enrolled in NCTN trials of commercially available agents between January 2013 and August 2017 were included if a complete medication list was available. Complete medication lists were collected from the date of enrollment or the next available date then screened utilizing protocol guidance and the DDI screening tool, Lexicomp® Drug Interactions (Wolters Kluwer, Hudson, OH). Interactions were reviewed for clinical relevance: defined as a DDI that would require a medication change to ensure study agent safety and efficacy at enrollment.

**Results:**

One hundred and twenty-eight subjects enrolled in 35 clinical trials were included. Protocol guidance detected 15 unique DDI pairs that should be avoided or used with caution in 10.2% (13/128) of subjects. The majority of these subjects did not have a clinically relevant DDI (69.2%, 9/13) based on pharmacist review. Lexicomp® detected moderate to major DDIs in 24.2% (31/128) of subjects, with 9.4% (12/128) having a clinically relevant DDI.

**Conclusions:**

This study confirms a high prevalence of DDIs present in subjects enrolling in oncology clinical trials. Further efforts should be made to improve methods to detect and manage DDIs in patients enrolling on clinical trials to ensure patient safety and trial data validity.

**Electronic supplementary material:**

The online version of this article (10.1186/s12885-018-5076-0) contains supplementary material, which is available to authorized users.

## Background

Clinically significant drug-drug interactions (DDIs) occur when one drug impacts the efficacy or safety of another drug taken concomitantly. Both drug and patient characteristics contribute to the risk for DDI. Drugs that are dependent on a single metabolic pathway (i.e. CYP3A4) and those that induce or inhibit commonly used metabolizing enzymes, such as CYP450, are more likely to cause DDI [1]. Additional mechanisms of DDIs include inhibition or induction of transporters, such as P-glycoprotein (P-gp), competitive protein binding, or modification of gastric acid leading to changes in medication absorption [2]. DDIs can also occur when two drugs taken together have opposing mechanisms of action (i.e. antihypertensive and stimulant), similar therapeutic effects (i.e. additive anticoagulation) or overlapping side effect profiles (i.e. QT prolongation, CNS depression, or immunosuppression) [3]. Many anti-cancer medications that are taken to prolong life have the potential to cause severe toxicity, consequently, small differences in drug concentration can determine which of these effects a patient experiences [1]. This makes it is critically important to identify and resolve potential DDI in oncology patients [2, 4]. In addition to medication characteristics, patient factors can also make DDI more likely to occur. Elderly patients comprise the majority of cancer diagnoses [5] increasing the likelihood of concomitant disease states managed by medications. Consequently, cancer patients have high rates of polypharmacy, which increases occurrence of DDI [6].

Despite standard DDI screening procedures, including pharmacist review of medication orders prior to dispensing and availability of DDI screening tools [7], clinically relevant DDIs are found in 16–41% of oncology patients [8–11]. This high prevalence suggests current DDI screening processes in oncology clinical care are suboptimal. This situation is likely even worse in oncology clinical trial subjects, for whom standard DDI procedures and tools do not exist. While systematic, pharmacist-conducted DDI screening for oncology clinical trial enrollment would be ideal [12], we have recently reported that many cancer centers do not systematically conduct DDI screening and that pharmacists are rarely involved, with DDI screening instead being the responsibility of clinical research coordinators or research nurses [13]. These individuals, who often lack DDI screening expertise [14, 15], rely primarily on DDI information contained in trial protocols.

The identification and management of DDIs for oncology clinical trial subjects is especially important because in addition to the impact of DDI on treatment safety and efficacy, DDI can also affect the accuracy and validity of data collected within the clinical trial. DDI could impact data validity by causing increased adverse outcomes due to added toxicity or decreased efficacy if study medication concentrations are impacted. Minimal research has been conducted to determine the prevalence of DDI in oncology clinical trial subjects to determine whether current DDI screening practices are sufficient.

National Clinical Trials Network (NCTN) clinical trials typically use commercially available oncology agents making it an ideal patient population to study based on the availability of DDI information. The objective of this study was to determine the prevalence of DDI involving study medications in subjects enrolled on NCTN clinical trials at the University of Michigan (U-M) Rogel Cancer Center. DDI were evaluated based on the study protocol’s recommendations for concomitant medication use (i.e. exclude, avoid, or use caution) and, separately, screening via a DDI tool (Lexicomp® Online, Wolters Kluwer, Hudson, OH). DDI identified using either technique were then manually assessed for clinical relevance by a pharmacist.

## Methods

### Protocol selection and patient data collection

All NCTN protocols of commercially available oncology agents (phase II through IV trials) that were open between January 2013 and August 2017 at the cancer center were included. Two protocols included medications that were approved during the trial, and subjects were excluded if they were enrolled prior to approval. Subjects were included if their complete medication lists on the date of enrollment or within one month of enrollment were available in the electronic medical record. Complete medication lists from each subject were recorded from the day of enrollment or the next available date. This study was granted exemption by the University of Michigan IRB.

### Protocol guided DDI screening

Protocols were reviewed for any guidance on DDIs that indicated the medication combination would cause trial exclusion or suggested the combination should be avoided or used with caution. Medication lists were screened for DDIs as defined by each respective protocol.

### Lexicomp® guided DDI screening

Medication lists were screened for major/contraindicated (level D or X) DDIs involving the study agent using Lexicomp® Drug Interactions. Lexicomp® was chosen based on our previous work examining DDI screening tool abilities to detect clinically relevant DDI [7]. Study medications were examined for the following medication characteristics based on the DDI mechanisms identified: P-gp transport, CYP450 metabolism, inhibition or induction, QT prolongation, CNS depression, protein binding, immunosuppression, anticoagulation and pH dependent absorption.

### DDI identification by pharmacist review

DDIs identified by the protocol guidance or Lexicomp® were reviewed by two individuals, a pharmacist and a Doctor of Pharmacy student, for clinical relevance. Clinical relevance was defined as a DDI that, in their judgement, would warrant a medication change prior to enrollment to ensure drug safety and/or efficacy based on currently available evidence (i.e. journal articles, FDA package inserts). Discordant decisions were discussed until consensus was reached.

### Protocol guidance assessment for identified clinically relevant DDI

Protocols were reviewed retrospectively for guidance regarding all clinically relevant DDI to determine the proportion of clinically relevant DDI for which there is no guidance in the clinical trial protocol.

### Statistical analysis

Descriptive statistics were calculated to determine the prevalence of DDI based on protocol guidance (exclude, avoid, and use with caution) or Lexicomp® (level D or X) and the rates of clinically relevant DDIs based on pharmacist review. The prevalence of clinically relevant DDI was also stratified by DDI mechanism (i.e. CYP450 metabolism, P-gp transport, pH dependent absorption, QT prolongation). Mechanisms were not included in the stratification if no clinically relevant interactions were found (i.e. anticoagulation, protein binding, immunosuppression, CNS depression). Additionally, the proportion of clinically relevant DDIs that impacted toxicity or efficacy of the study agent, as opposed to a concomitant medication, was determined. An independent samples t-test was performed to compare the number of concomitant medications taken by subjects who did and did not have a clinically relevant DDI, based on pharmacist review. IBM® SPSS® Statistics (v24) software was used for the statistical analysis.

## Results

### Enrolled subject characteristics

**Fig. 1:**
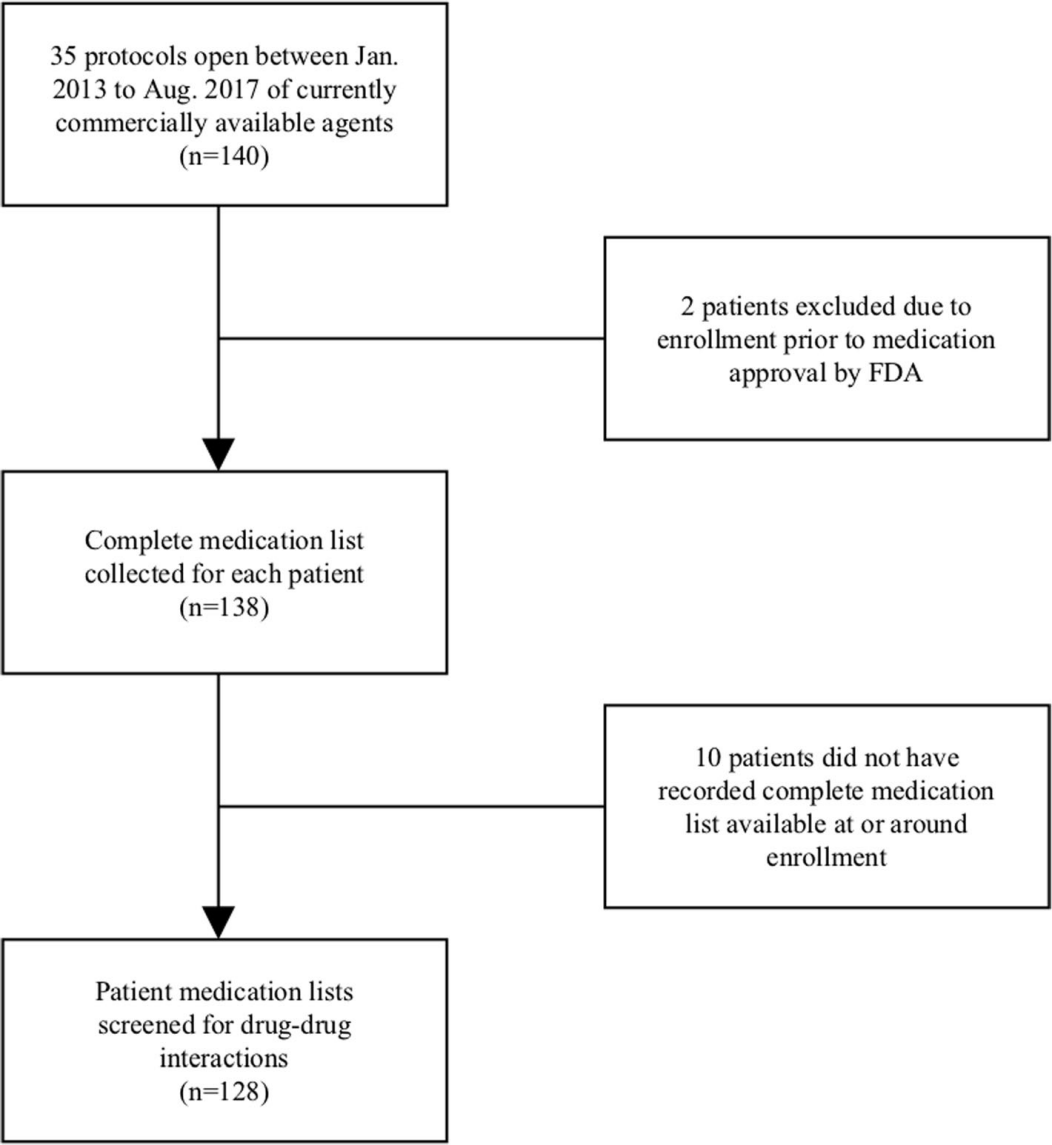
Flow diagram indicating patient exclusion criteria. Protocols were included if they exclusively used medications that were commercially available, however, patients were excluded if they were enrolled prior to FDA approval of the agents (*n* = 2, 1.4%). Patients were excluded if a complete medication list was not available at or around the time of enrollment (*n* = 10, 7.1%)

One hundred and twenty-eight patients were included in the analysis (Fig. 1) from 35 NTCN protocols including mostly monoclonal antibodies and protein kinase inhibitors (Additional file 1). Ten subjects were not included because a complete medication list was not available. Enrolled subjects were taking an average of 6.8 concomitant medications (standard deviation: 4.77).

### DDIs detected by Lexicomp®

**Fig. 2:**
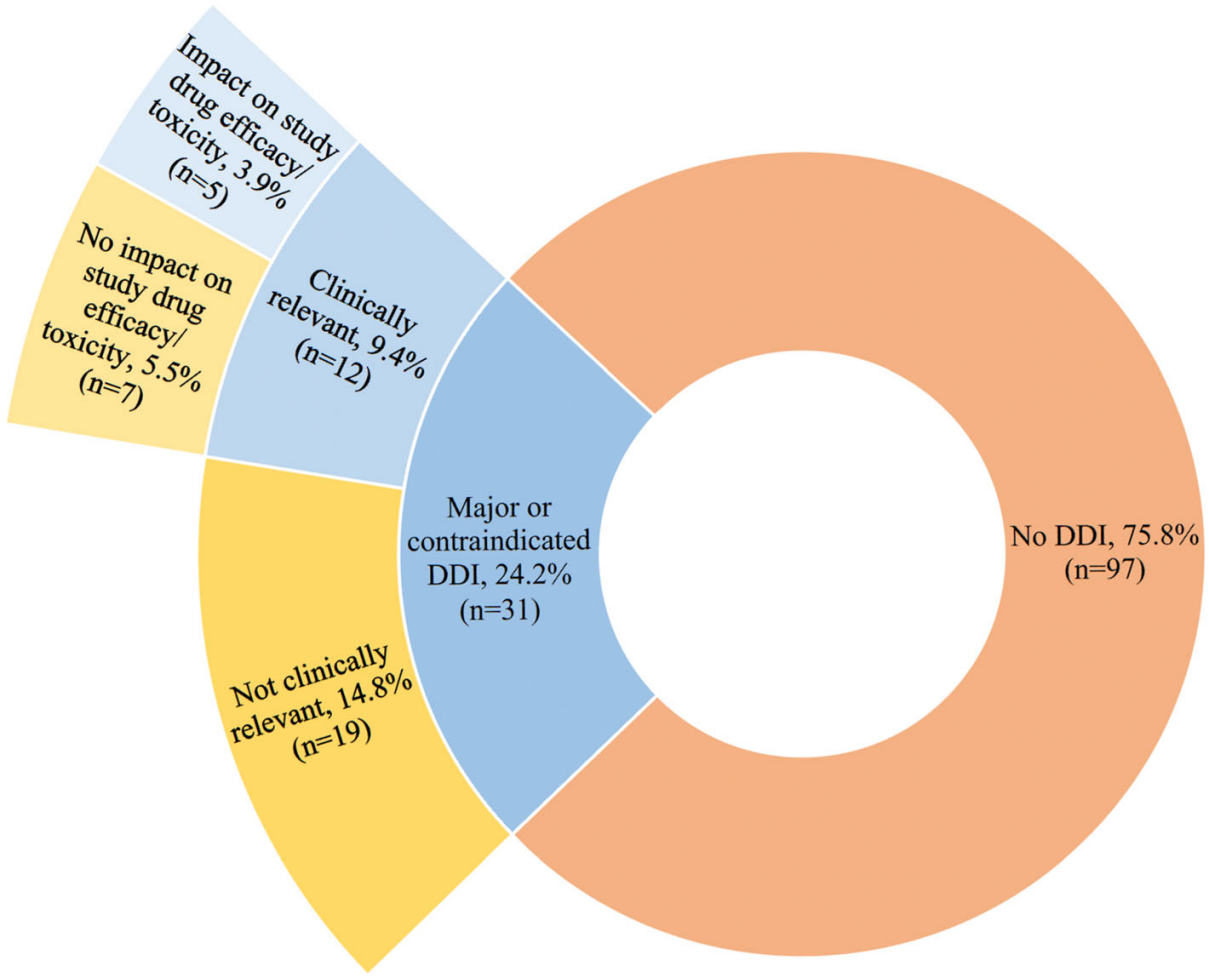
Breakdown of Lexicomp® detected drug-drug interactions by patient and their clinical relevance. Patients were screened for major and contraindicated drug-drug interactions with Lexicomp® Drug Interactions. The major and contraindicated interactions detected were examined for clinical relevance by patient, and then further examined if they would impact study agent efficacy or toxicity. A total of 3.9% patients (*n* = 5) had clinically relevant interactions that could impact the study agent efficacy or toxicity in addition to validity of study data. DDI = drug-drug interaction

Lexicomp® detected 43 DDIs in 31 (24.2%) subjects with a range of 0–3 DDIs per patient. Pharmacist review of the clinical relevance of DDI detected by Lexicomp identified 10 unique DDI pairs, which occurred 13 times in 12 (9.4%) unique patients (Table 1). Only one subject had two clinically relevant DDIs. Subjects with a clinically relevant DDI were taking a greater mean number of concomitant medications than those without a clinically relevant DDI (9.33 vs. 6.50, 95% CI: 0.006 to 5.661, *p* = 0.05). Of the 12 patients with clinically relevant DDIs, the efficacy or toxicity of the study agent was affected in five subjects (41.7%, 5/12, Fig. 2).

**Table 1:** Interactions Detected by Protocol Guidance and Lexicomp® Including Their Mechanism of Interaction and Clinical Relevance

Study Agent(s)	Interacting Drug (n with interaction)	Protocol Guidance	Lexicomp®	Clinically Relevant^a^	Interaction Mechanism
everolimus	carvedilol (2)	Avoid	yes	yes	P-gp
dasatinib	antacids (1)	Avoid	yes	yes	pH
vemurafenib	ondansetron (1)	Avoid	yes	yes	QT
dabrafenib	amlodipine (2)	Avoid	yes	no	CYP450
vemurafenib	venlafaxine (1)	Avoid	yes	no	QT
cabozantinib	ondansetron (1)	Avoid	no	no	QT
ipilimumab, nivolumab	ondansetron (2)	Avoid	no	no	QT
dabrafenib	omeprazole^b^ (1)	Caution	yes	no	pH
dabrafenib	atorvastatin (1)	Caution	yes	no	CYP450
dabrafenib	omeprazole^c^ (1)	Caution	yes	no	CYP450
olaparib	cetirizine (1)	Caution	no	no	P-gp
olaparib	ranitidine (1)	Caution	no	no	P-gp
testosterone	dabigatran (1)	Caution	no	no	anticoagulation
trametinib	duloxetine (1)	Caution	no	no	protein binding
trametinib	olanzapine (1)	Caution	no	no	protein binding
enzalutamide	amlodipine (2)	None	yes	yes	CYP450
enzalutamide	citalopram (1)	None	yes	yes	CYP450
enzalutamide	diltiazem (1)	None	yes	yes	CYP450
enzalutamide	omeprazole (1)	None	yes	yes	CYP450
enzalutamide	tramadol (2)	None	yes	yes	CYP450
enzalutamide	venlafaxine (1)	None	yes	yes	CYP450
crizotinib	escitalopram (1)	None	yes	yes	QT
dabrafenib	tamsulosin (1)	None	yes	no	CYP450
enzalutamide	alprazolam (1)	None	yes	no	CYP450
enzalutamide	hydrocodone (1)	None	yes	no	CYP450
enzalutamide	modafinil (1)	None	yes	no	CYP450
enzalutamide	ondansetron (2)	None	yes	no	CYP450
enzalutamide	tamsulosin (1)	None	yes	no	CYP450
dexamethasone	echinacea (1)	None	yes	no	immunosuppression
pomalidomide	echinacea (1)	None	yes	no	immunosuppression
pomalidomide	hydrocodone (3)	None	yes	no	CNS depression
pomalidomide	oxycodone (2)	None	yes	no	CNS depression
pomalidomide	tramadol (4)	None	yes	no	CNS depression
crizotinib	granisetron (1)	None	yes	no	QT
vemurafenib	albuterol (1)	None	yes	no	QT
dexamethasone	antacids (3)	None	yes	no	unknown

*P-gp* P-glycoprotein

^a^Clinical relevance was determined by study team review and defined as a drug-drug interaction that would require a medication change to ensure study agent safety/efficacy at enrollment

^b^dabrafenib as victim and omeprazole as perpetrator

^c^omeprazole as victim and dabrafenib as perpetrator

The most prevalent medication characteristic was CYP450 metabolism, and 7.1% (7/98) of subjects receiving a study agent that had CYP450 DDI potential had a clinically relevant DDI (Table 2). All of the clinically relevant DDI with CYP450 DDI potential were through CYP3A4.

**Table 2:** Number of Clinically Relevant Interactions Stratified by Medication Characteristics

Medication Characteristic	Total patients on protocol with characteristic	Clinically relevant^a^ interactions with characteristic (%)
CYP450 metabolism	98	7 (7.1%)
P-glycoprotein transport	69	2 (2.9%)
QT prolongation	23	2 (8.7%)
pH dependent absorption	6	1 (16.7%)

^a^Clinical relevance was determined by study team review and defined as a drug-drug interaction that would require a medication change to ensure study medication safety/efficacy at enrollment

### DDIs detected by protocol guidance

**Fig. 3:**
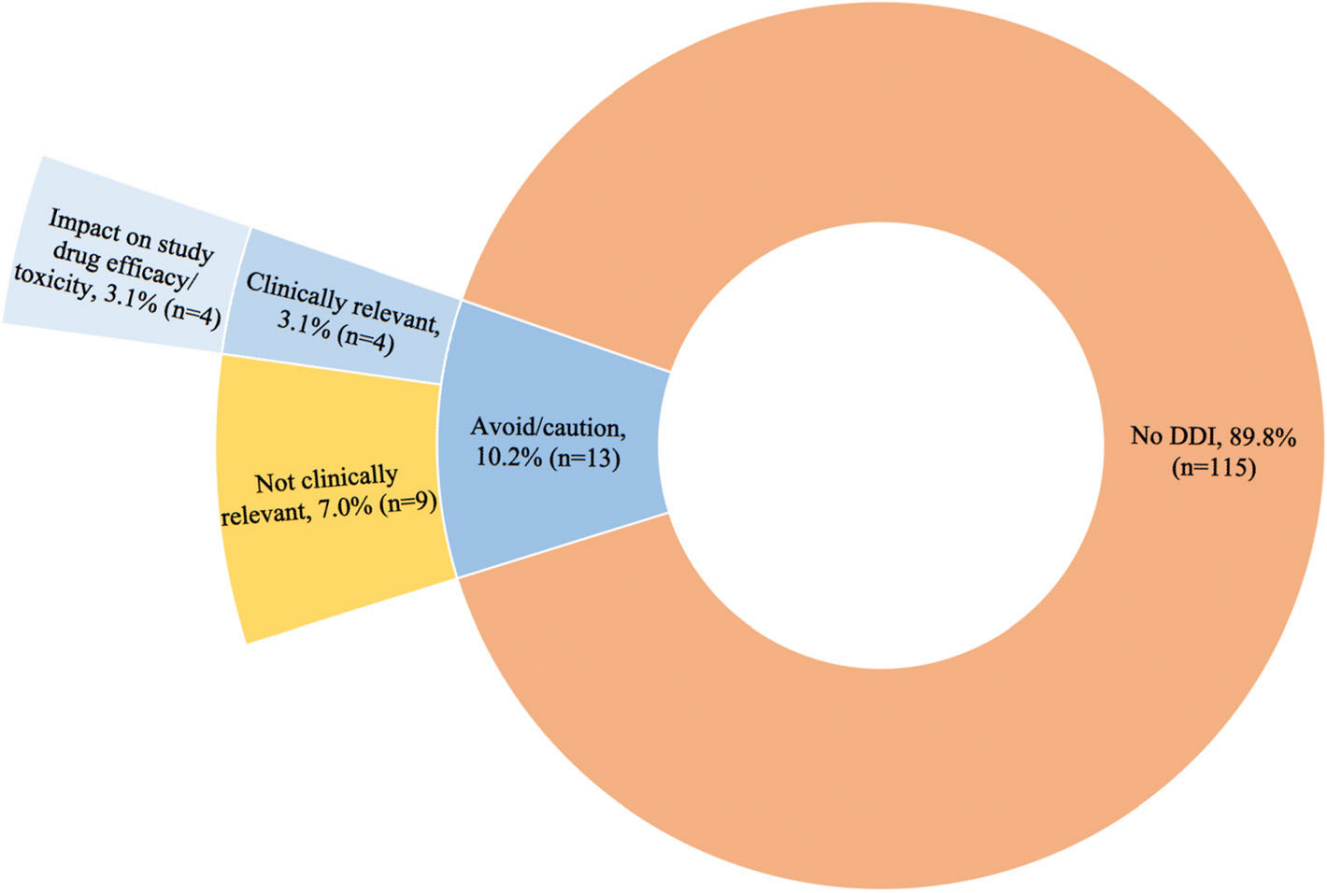
Breakdown of protocol guidance detected drug-drug interactions by patient and their clinical relevance. Patients were screened for drug-drug interactions based on protocol guidance to exclude, avoid or use medications with caution. Interactions were examined for clinical relevance by patient, and then further examined if they would impact study agent efficacy or toxicity. A total of 10.2% patients (*n* = 13) were taking a concomitant medication that was suggested to use with caution or avoid with the study agent, and 3.1% (*n* = 4) had a clinically relevant interaction. DDI = drug-drug interaction

The majority of protocols (26/35, 74.3%) included guidance to exclude, avoid or use concomitant medications with caution. Seven protocols included guidance to use a concomitant medication with caution (7/35, 20.0%), 17 included DDI to avoid (17/35, 48.6%), and 16 included DDI that would exclude a subject from enrollment (16/35, 45.7%). Based on protocol guidance, 17 total DDIs were detected in 13 (10.2%) patients with a range of 0–2 DDIs per subject (Fig. 3). No patients were taking medications that were considered protocol exclusion criteria, 13 (10.2%) subjects were taking medications that were suggested by the protocols to be avoided if possible or used with caution. Of the 15 unique DDI pairs detected based on protocol guidance, 3 (20.0%) interactions were considered clinically relevant based on the pharmacist review (Table 1) and occurred in 4 subjects (3.1%). Of the subjects with clinically relevant DDI, all of the DDI would have impacted the efficacy or toxicity of the study agent.

### Retrospective assessment of protocol guidance for clinically relevant DDI

Of the 10 DDI determined to be clinically relevant by pharmacist review, 3 (30%) were suggested to avoid in the study protocols. The remaining 7 (70%) were not mentioned in the protocols; none of the 10 clinically relevant DDIs were considered exclusion criteria in the protocols.

## Discussion

Oncology patients are at a higher risk of DDI due to their high rates of polypharmacy [6] and the sensitivity of efficacy and toxicity to small changes in the concentration of anti-cancer drugs [1]. Although DDI risk has been assessed in oncology patients, it has not been extensively examined in clinical trial subjects. It is extremely important in this population to manage DDI because they not only impact subject safety and efficacy but also trial data accuracy. The objective of this study was to determine the proportion of subjects enrolling in NCTN trials at the U-M Rogel Cancer Center with DDI detected based on protocol guidance, Lexicomp® screening, and manual pharmacist review.

Screening by protocol guidance identified DDIs in 10.2% of patients and Lexicomp® identified major or contraindicated DDI in 24.2% of subjects. Only one prior retrospective DDI study has been conducted in oncology trial subjects to the authors’ knowledge. Phase I oncology clinical trial subjects were examined for CYP450 DDIs prior to and after enrollment. Prior to enrollment, 69% of subjects had at least one DDI with the study agent [16]. The majority (85%) of these DDI were resolved during screening by discontinuing or switching the interacting medications, while the remaining 15%, which were not listed as exclusion criteria in the protocol, were unresolved after enrollment [16]. Similarly, none of the clinically relevant DDIs identified in subjects enrolled on NCTN trials in our analysis were considered exclusions to enrollment.

The prevalence identified in our study (9.4–24.2%) is on the lower end of DDI rates found in oncology patients (16–41%) [8–11]. The higher DDI rates in some studies could be attributed to inclusion of DDIs between concomitant medications or supportive care agents in addition to DDI with anticancer agents or inclusion of less severe DDI [10, 11]. The finding that subjects with a clinically relevant DDI were taking a greater number of concomitant medications (6.50 vs. 9.33) is consistent with previous reports that oncology patients taking more concomitant medications have higher rates of DDI [6, 8].

Not all DDIs identified by protocol guidance or Lexicomp® would have posed an immediate risk to the subject. After examining the DDIs flagged by protocol guidance and Lexicomp® for clinical relevance, 9.4% of subjects in this study had an DDI that, in our judgement, would have required immediate action at enrollment to ensure patient safety or efficacy. About half of these DDIs would have impacted efficacy or toxicity of the study agent, which, in addition to its importance in ensuring optimal treatment outcomes for the subject, would also affect the accuracy and validity of the clinical trial data. The majority of identified DDI can be easily resolved by dose changes [17], increased monitoring (e.g. QT prolongation) [18], or a medication change [19], and would not warrant trial exclusion.

Only 20.0% of the DDI pairs identified based on protocol guidance were considered clinically relevant. Additionally, 70.0% of the clinically relevant DDI pairs were not identified by the protocols suggesting that protocol guidance may not be optimally calibrated for DDI screening. Pharmacist screening is standard of care in clinical practice, and pharmacist conducted medication reviews would be ideal for trial enrollment [12]. However, pharmacists are not always available and are not typically involved in screening of clinical trial subjects [13]. The frequency of clinically relevant DDI in subjects enrolled on NCTN trials indicates improvements can be made to reduce DDI. Possible solutions include improving protocol guidance to encompass more clinically relevant DDI and improving the DDI screening process to ensure oncology clinical trial subject safety, efficacy, and data accuracy.

Currently there are DDI screening tools available for clinical practice, but none have been developed for clinical trial enrollment. DDI screening tools available for use in clinical practice are not ideally suited for use in oncology clinical trial screening. These tools only include commercially available agents, often contain technical language, and identify DDI between all drugs a patient is on [20, 21]. A DDI screening tool that includes investigational agents, provides alerts using language suited for multiple levels of health care providers, only identifies DDIs involving the study agent, and filters medication characteristics based on protocol guidance would be optimal for DDI screening in oncology clinical trials.

This retrospective analysis was not able to determine whether any DDI were detected and resolved during screening, therefore, we have limited ability to assess the frequency or effectiveness of DDI screening within our cancer center. Additionally, the clinical outcome of the DDI was not assessed in this cohort, and it is unknow if the detect DDI caused adverse patient outcomes. It is possible that medication lists were not completely accurate, although it is standard procedure at U-M to update medication lists at the start of all patient visits. Also, a standardized approach was taken to determine which DDIs were clinically relevant; however, if a different pharmacist reviewed the DDIs, our results would likely have changed slightly. Finally, this study was a convenience sample conducted only at the University of Michigan Rogel Cancer Center for NCTN trials with a small number of subjects and these findings might not be generalizable to other institutions or non-NCTN oncology trials.

## Conclusion

This study indicates a high prevalence of clinically relevant DDI in subjects enrolling on NCTN clinical trials. Further efforts should be made to detect and manage DDI, potentially through improvements in protocol guidance, pharmacist involvement in screening, and development of a clinical trial-specific DDI screening tool. Further research is needed to confirm that identifying clinically relevant DDI and resolving them prior to clinical trial enrollment would improve patient outcomes and ensure validity of data collected within clinical trials.

## Additional file

Additional file 1: Protocol medications by class. The included protocol medications listed by medication class. (DOCX 14 kb)

## Abbreviations

DDI: Drug-drug interaction
NCTN: National Clinical Trials Network
P-gp: P-glycoprotein
