# Supplementary material for: Prevalence of drug-drug interactions in oncology patients enrolled on National Clinical Trials Network oncology clinical trials
Source: BMC Cancer. 2018 Nov 22;18:1155. doi: 10.1186/s12885-018-5076-0 (PMC6249716; doi:10.1186/s12885-018-5076-0)
Supplement: Supplementary file 1 — Protocol medications by class. The included protocol medications listed by medication class. (DOCX 14 kb) [file 12885_2018_5076_MOESM1_ESM.docx]

**Additional file 1. Protocol medications by class**

| **Class** | **Medications** |
| --- | --- |
| Protein kinase inhibitors | afatinib, axitinib, cabozantinib (2), crizotinib (2), dabrafenib (2), dasatanib, everolimus (2), pazopanib, sorafenib, temsirolimus, trametinib |
| Monoclonal antibodies | bevacizumab, blinatumomab (2), brentuximab (2), cetuximab (2), dinutuximab, durvalumab, ipilimumab (5), nivolumab (3), pembrolizumab, ramucirumab, trastuzumab |
| Antimetabolite | flurouracil, gemcitabine, methotrexate, mercaptopurine |
| Alkylating agent | cyclophosphamide, oxaliplatin, temozolomide |
| Proteasome inhibitor | bortezomib (2), carfilzomib, ixazomib |
| Topoisomerase inhibitor | doxorubicin, irinotecan (2), topotecan |
| Antimicrotubular | paclitaxel, vincristine |
| Corticosteroid | dexamethasone (2), prednisone |
| Hormonal therapy | enzalutamide, testosterone |
| Immunomodulator | lenalidomide (2), pomalidomide |
| PARP inhibitor | olaparib |
| Other | celecoxib, dexrazoxane, interferon alfa-2b, leucovorin |

(N) the number of protocols that include that medication
